# Midbrain Organoids: A New Tool to Investigate Parkinson’s Disease

**DOI:** 10.3389/fcell.2020.00359

**Published:** 2020-05-19

**Authors:** Lisa Maria Smits, Jens Christian Schwamborn

**Affiliations:** Luxembourg Centre for Systems Biomedicine, University of Luxembourg, Belvaux, Luxembourg

**Keywords:** midbrain, organoid, Parkinson’s disease, self organization, stem cell research

## Abstract

The study of human 3D cell culture models not only bridges the gap between traditional 2D *in vitro* experiments and *in vivo* animal models, it also addresses processes that cannot be recapitulated by either of these traditional models. Therefore, it offers an opportunity to better understand complex biology including brain development. The brain organoid technology provides a physiologically relevant context, which holds great potential for its application in modeling neurological diseases. Here, we compare different methods to obtain highly specialized structures that resemble specific features of the human midbrain. Regionally patterned neural stem cells (NSCs) were utilized to derive such human midbrain-specific organoids (hMO). The resulting neural tissue exhibited abundant neurons with midbrain dopaminergic neuron identity, as well as astroglia and oligodendrocyte differentiation. Within the midbrain organoids, neurite myelination, and the formation of synaptic connections were observed. Regular neuronal fire patterning and neural network synchronicity were determined by multielectrode array recordings. In addition to electrophysiologically functional neurons producing and secreting dopamine, responsive neuronal subtypes, such as GABAergic and glutamatergic neurons were also detected. In order to model disorders like Parkinson’s disease (PD) *in vitro*, midbrain organoids carrying a disease specific mutation were derived and compared to healthy control organoids to investigate relevant neurodegenerative pathophysiology. In this way midbrain-specific organoids constitute a powerful tool for human-specific *in vitro* modeling of neurological disorders with a great potential to be utilized in advanced therapy development.

## Introduction

The development of the organoid methodology counts today as a major technological breakthrough in stem cell research. It enabled immense advances in the application of human induced pluripotent stem cells (hiPSCs) and was even celebrated as the “Method of the Year” in 2017 (“Lancaster et al., 2013”).

## Cerebral Organoids

Initial experiments on self-organization of PSCs under 3D conditions were performed more than 10 years ago. Yet, it were the findings of Lancaster et al., that actually launched a new era in research on the human brain by introducing the “cerebral organoids” in 2013 (Lancaster et al., 2013; Kelava and Lancaster, 2016b). Supporting the conclusions of initial 3D culture experiments, Lancaster et al. took advantage of the PSCs’ intrinsic self-organization and derived neuroepithelium under 3D conditions (Kadoshima et al., 2013). To avoid limitations on specific brain region identities, they did not add patterning growth factors. Instead, after aggregation, the cells were embedded in Matrigel, a surrogate matrix that was previously introduced for the generation of intestinal organoids (Sato et al., 2009). This serves as a structural support, inducing the correct polarity stimulus to promote the complex outgrowth of large, apicobasal neuroepithelial buds (Lancaster and Knoblich, 2014a; Wang X. et al., 2018). These buds expand during the course of culture and acquire not only various brain identities but also fluid-filled lumina reminiscent of brain ventricles. To improve nutrient supply and oxygen exchange, the floating cerebral organoids were cultured in spinning bioreactors or on orbital shaker plates, allowing organoids to grow up to 4 mm in diameter (Lancaster and Knoblich, 2014a; Kelava and Lancaster, 2016b). These optimized growth conditions combined with the intrinsic self-organizing capabilities of PSCs resulted in the formation of a variety of brain regions within a single organoid, including hindbrain, midbrain, forebrain, and even retinal tissue identities (Lancaster et al., 2013; Lancaster and Knoblich, 2014b; Renner et al., 2017; Wang X. et al., 2018). Remarkably, a detailed study about the patterning events during the course of cerebral organoid development and differentiation indicates that spatial and temporal patterning events are reminiscent of those determining the human brain development (Renner et al., 2017).

## Modification of Cerebral Organoids

The classic cerebral organoid protocol, describing the generation of general whole-brain organoids, has been modified by many research groups in the last years and has resulted in the formation of more regionally specific 3D cell cultures (Lancaster and Knoblich, 2014a; Lancaster et al., 2016). In the study of Paşca et al., 2015), both bone morphogenetic protein (BMP) and transforming growth factor β (TGF-β) signaling pathways were inhibited by the small molecules Dorsomorphin (DM) and SB-431542 (SB) to achieve an effective neural induction. This dual-SMAD inhibition, in combination with fibroblast growth factor (FGF) 2, epidermal growth factor (EGF) and the absence of extracellular scaffolding, gave rise to neural progenitors expressing the dorsal telencephalic markers paired box protein 6 (PAX6) and forkhead box protein G1 (FOXG1). Further neuronal differentiation was promoted by replacing FGF2 and EGF with brain-derived neurotrophic factor (BDNF) and neurotrophic factor 3 (NT3) and led to the generation of various neural and glial identities of the dorsal cortex within each spheroid, including superficial and deep cortical layer neurons (Paşca et al., 2015; Kelava and Lancaster, 2016b). With this, Paşca et al. described a method that gave rise to a 3D culture specific of a brain subregion; a culture that exhibited a reduced number of ectodermal derivatives compared to the original cerebral organoid protocol. A similar approach of using a dual-SMAD inhibition was published by Qian et al. (2016). While maintaining the basis of the LANCASTER protocol, such as Matrigel embedding and agitation, they demonstrated that it is possible to culture organoids in 3D printed miniaturized bioreactors, thus enabling more feasible, scaled-up productions of neural 3D cultures (Qian et al., 2016). Another advantage of the self-engineered multi-well spinning device is the possibility of comparing numerous different culture conditions in parallel. Paşca et al. aimed to reduce the tissue heterogeneity of the cerebral organoids and therefore pre-patterned the embryoid bodies (EBs) to obtain specific brain regions. The inhibition of TGF-β signaling by SB and activation of Wnt signaling by glycogen synthase kinase 3 (GSK-3β) inhibitor CHIR-99021 (CHIR) within the first 2 weeks of culture, resulted in forebrain organoids organized in defined, multi-layered progenitor zones, including homologs to the ventricular zone (VZ), the inner and outer subventricular zones (SVZ). Moreover, neuronal types of all six cortical layers could be detected within these forebrain-specific organoids. With the help of the mini-bioreactors, Qian et al. (2016) also developed a method to derive hypothalamic-specific organoids. After a dual-SMAD inhibition with SB and LDN-193189 (LDN), they patterned the neuroectodermal cells to a hypothalamic fate by activating Wnt and sonic hedgehog (SHH) signaling, applying WNT3a, SHH, and Purmorphamine (PMA) to the culture. 40 days later, these organoids contained cell populations expressing markers specific of hypothalamic neuronal lineages (Qian et al., 2016).

## Disease Modeling With Brain Organoids

Similar characteristics on a molecular, cellular and physiological basis between human brain organoids and the actual human brain justify their increasing application in studying brain biology and modeling neurological disorders (Wang Y. et al., 2018). Already Lancaster et al. (2013) discovered the potential of cerebral organoids as a model to detect impaired neurodevelopment and they derived organoids carrying a mutation that causes microcephaly. It was also suggested that a Zika virus (ZIKV) infection was causing microcephaly in neonates. In 2016, the World Health Organization (WHO) declared the ZIKV and its associated complications an emergency of public health (Qian et al., 2016; Dutta et al., 2017). This activation of the global research community led to an accelerated development of vaccines and treatments, with many studies based on cerebral organoids, which were able to recapitulate features of human cortical development *in vitro* (Cugola et al., 2016; Dang et al., 2016; Garcez et al., 2016; Miner and Diamond, 2016; Nowakowski et al., 2016; Qian et al., 2016; Wells et al., 2016; Xu et al., 2016). The results of these studies indicate that a ZIKV infection affects the neurogenesis, disrupts the cortical layers of the organoids, and, in a similar manner, causes microcephalic-like deficits in cortical development (Dutta et al., 2017). Due to the specific embryonic formation of human brains, only a human-specific 3D cell culture model exhibiting advanced organizational features could have led to the reported discoveries. Neither murine nor 2D cell culture were able to address the potential link between ZIKV and microcephaly (Qian et al., 2016; Dutta et al., 2017; Setia and Muotri, 2019). In addition to this successful application, brain organoids have proven useful to study other neurological disorders. Recently, so-called “tumouroids” have been established from human glioblastoma, the most common and aggressive brain cancer (Dutta et al., 2017). The hypoxic gradients and stem cell heterogeneity found in these tumouroids cannot be recreated via conventional culture methods. Therefore, glioblastoma organoids offer a unique opportunity for their application in brain cancer diagnostics and therapeutics (Hubert et al., 2016; Dutta et al., 2017; Bian et al., 2018). Furthermore, two different approaches using 3D human neural cell culture systems were reported to recapitulate Alzheimer’s disease (AD) phenotypes *in vitro* (Choi et al., 2014; Raja et al., 2016). These 3D cultures provide an environment that promotes the formation of amyloid-β (Aβ) plaques and neurofibrillary tangles (NFTs), pathological events that could not have been serially linked before by using 2D cultured human neurons (Choi et al., 2014, 2016; D’Avanzo et al., 2015; Raja et al., 2016). This confirms that the evolving brain organoid methodology facilitates the development of more precise human cellular models that can support the research of neurodegenerative disorders. The technology of more complex 3D cell culture systems not only bridges the gap between traditional 2D *in vitro* experiments and *in vivo* animal models, but also addresses processes that cannot be recapitulated by these traditional models. For example, drug failure or unanticipated side-effects upon translation to humans can be a result of the different metabolisms of humans and animals. Therefore, human organoids offer an opportunity to unravel complex biological processes, such as the development of the human brain, where conventional models have not proven successful. The establishment of stem cell-derived brain organoids allows the modeling of key aspects of human brain development *in vitro*, by utilizing the enormous differentiation potential of PSCs and their ability to self-organize with a specific spatial orientation. Overall, this novel technology provides a physiologically relevant context, such as interactions between glia cells and neurons in a spatially organized microenvironment, which holds great potential for its application in modeling neurological diseases.

## Discussion

The lack of advanced experimental *in vitro* models that truly recapitulate the complexity of the human brain is one of the main limitations in neuroscience and in the field of disease modeling. Current *in vitro* approaches to model physiology and pathology of human neurons are primarily based on cultures of neurons grown under 2D conditions. While the resulting monolayer cell cultures have proven useful as a tool to study disease mechanisms and to identify potential neuroprotective compounds (Nguyen et al., 2011; Cooper et al., 2012; Sánchez-Danés et al., 2012; Reinhardt et al., 2013b; Ryan et al., 2013; Qing et al., 2017; Spathis et al., 2017), these culture conditions do not model several characteristics which are relevant to the human brain. Features such as cell-cell interactions and cytoarchitecture might be crucial to predict the effectiveness of *in vitro* tested compounds in clinical trials (Abe-Fukasawa et al., 2018). In this case, the *in vitro* human brain organoid technology is a valuable tool, it allows to opportunity to understand complex biology in a physiologically relevant context and also enables advances in translational applications (Fatehullah et al., 2016). Originally, brain organoid approaches relied on the endogenous capacity of PSCs to self-organize under 3D conditions, intrinsically following early steps of the brain development (Arlotta, 2018). These approaches resulted in ectodermal derivatives with complex cytoarchitectures beyond what is possible with 2D PSC derivatives (Kadoshima et al., 2013; Lancaster et al., 2013; Paşca et al., 2015). Since neurons form functional networks with other neurons and non-neuronal cells in the brain, it is essential to expand the research of neurodegenerative diseases by exploiting 3D models that are able to reproduce these interactions. In general, 3D conditions are able to more closely mimic *in vivo* environments and therefore enable an accelerated neuronal differentiation and network formation *in vitro* (Haycock, 2011; D’Avanzo et al., 2015). Moreover, it has been shown that neurons developed in a 3D environment express a more representative range of neuronal genes than neurons derived in 2D conditions (Seidel et al., 2012). A monolayer of neurons cannot provide as many connections between individual cells as a 3D neuronal culture, and the smaller synaptic distances in a 3D neuronal network promotes functional signal transduction (Cullen et al., 2012; D’Avanzo et al., 2015). By creating a third dimension, neurons develop in an environment that is closer to nature and actually relevant to human physiology, consequently gaining morphological and physiological properties similar to those *in vivo*.

Specifically, the generation and characterization of a novel midbrain-specific 3D cell culture system provides an advanced *in vitro* model to study neurodevelopmental processes as well as neurodegenerative diseases of the human midbrain. In order to achieve the formation of these highly specialized structures, distinctively resembling the human midbrain, organoids have been derived from regionally patterned neural stem cells (NSCs). This particular starting population, already committed to the ventral neural tube fate of the mesencephalon with further application of spatio-temporal specific signaling under 3D culture conditions, has led to the establishment of novel human midbrain-specific organoids (hMO). Here, we compare and evaluate newly derived hMO methods that create a powerful tool for human-specific *in vitro* disease modeling of neurological disorders.

## Derivation of Midbrain-Specific Organoids

To achieve the *in vitro* derivation of the human midbrain, additional stimuli of specific pathways along with the 3D PSC culture are required. To date, six 3D cell culture approaches have been published by different research groups for deriving tissue that resembles features of the human midbrain (Tieng et al., 2014; Jo et al., 2016; Qian et al., 2016; Monzel et al., 2017; Kim et al., 2019; Smits et al., 2019b). All of these approaches can be attributed to previous 2D cell culture experiments, which explored the fundamental principles for the generation and characterization of midbrain fate-specific cells, derived from PSC via exogenous patterning cues (Kriks et al., 2011; Kirkeby et al., 2012; Reinhardt et al., 2013a). For instance, Qian et al. were inspired by 2D experiments performed by Kriks et al. (2011) and also initial midbrain-like tissue experiments reported by Tieng et al. (2014) and (Qian et al., 2016). In the same year, another protocol describing the generation of hMOs was published (Jo et al., 2016). Jo et al. (2016) and later Kim et al. (2019) based their derivation on the findings of Chambers et al. (2009). Additionally, hMO protocols described by Monzel et al. (2017) and Smits et al. (2019b) are based on 2D experiments by Reinhardt et al. (2013a).

The initial step for the derivation of hMOs is the formation of EBs and induction of the neuroectoderm via dual-SMAD inhibition. In all hMO protocols compared here, SB was used to inhibit the Activin/TGF-β signaling pathway (Table 1). The combination with BMP pathway inhibitors enabled a full neural conversion of the PSCs. The use of BMP antagonists Noggin, LDN, and DM has been shown in 2D (Chambers et al., 2009; Kriks et al., 2011; Reinhardt et al., 2013a) and applied in 3D stem cell cultures (Table 1). To control the specification of the neural progenitor cells, CHIR, a potent chemical inhibitor of GSK-3β, was used to dose-dependently activate the WNT signaling pathway (Kirkeby et al., 2012). The final patterning, toward midbrain floor plate precursors, requires a treatment with small molecule activators of the SHH signaling pathway, such as recombinant SHH, PMA or smoothened agonist (SAG) (Kriks et al., 2011; Smits et al., 2019b). This composition of exogenous patterning cues results in neural progenitors that can give rise to authentic, midbrain-specific dopaminergic neurons (mDANs) (Kriks et al., 2011; Kirkeby et al., 2012; Doi et al., 2014; Nolbrant et al., 2017).

**TABLE 1:** Comparison of hMO derivation protocols: Overview of applied compounds to derive midbrain-specific neuralectoderm by neural induction.

	**Tieng et al. (2014)**	**Qian et al. (2016)**	**Jo et al. (2016)**	**Monzel et al. (2017)**	**Kim et al. (2019)**	**Smits et al. (2019b)**
**Dual-SMAD inhibition**						
SB	10 μM	10 μM	10 μM	10 μM	10 μM	10 μM
Noggin	–	–	200 ng/ml	–	200 ng/ml	–
LDN	100 nM	100 nM	–	–	–	150 nM
DM	–	–	–	1 μM	–	–
**WNT activation**						
CHIR	3 μM	3 μM	3 μM	3 μM	3 μM	3 μM
**SHH activation**						
SHH	100 ng/ml	100 ng/ml	100 ng/ml	–	100 ng/ml	–
PMA	2 μM	2 μM	–	0.5 μM	–	–
SAG	–	–	–	–	–	0.5 μM
**FGF8 activation**						
FGF8	100 ng/ml	100 ng/ml	100 ng/ml	–	100 ng/ml	–

*hMO protocols from Tieng et al. (2014) and Qian et al. (2016) are based on 2D experiments by Kriks et al. (2011); hMO protocols from Jo et al. (2016) and Kim et al. (2019) are based on 2D experiments by Chambers et al. (2009); hMO protocols from Monzel et al. (2017) and Smits et al. (2019b) are based on 2D experiments by Reinhardt et al. (2013a).*

The further development of these protocols allowed the derivation of 3D cultured hMO. In contrast to 2D monolayer cultures, hMOs can recapitulate complex interactions of mDANs with other cell types of the central nervous system (CNS) in a 3D environment. Cluster analysis, comparing the differentially expressed genes of hMOs, 2D-cultured mDANs, and human prenatal midbrain samples indicated that hMOs share features of gene expression profiles of the prenatal midbrain, which cannot be recreated via the conventional 2D derivation method for mDANs (Jo et al., 2016). This demonstrates that the specific cellular structure and heterogeneity of the midbrain-specific organoid cultures allows biological aspects to be modeled, which cannot be mimicked with current 2D stem cell cultures. Additionally, enormous amounts of disease-relevant mDANs can be produced in a rapid and reproducible way, which is required for disease modeling and drug discovery in the field of Parkinson’s disease (PD). Numerous published protocols describe the generation of ventral mDANs from human PSCs in 2D by replicating mDAN’s *in vivo*-specification *in vitro* (Kriks et al., 2011; Kirkeby et al., 2012; Doi et al., 2014; Nolbrant et al., 2017). Although current protocols are based on the generation of LMX1A/FOXA2 positive midbrain floorplate progenitors, differentiations starting from PSCs are time-consuming and typically result in cultures containing various neuronal identities (Hargus et al., 2010; Kriks et al., 2011; Kirkeby et al., 2012; Grealish et al., 2014). Typically, brain organoids are generated from PSCs by exploiting developmental processes (Lancaster et al., 2013; Clevers, 2016) or by creating an environment favoring specific stem cell niches (Tieng et al., 2014; Jo et al., 2016; Qian et al., 2016; Kim et al., 2019). However, the utilization of NSCs as a starting population for hMOs has the advantage that already patterned cells differentiate more efficiently into the desired structures. Other adult stem cell-derived organoid cultures have been established for instance, for the generation of intestinal or lung organoids. They contain cell types that are present in the organ from which they were derived and recapitulate some degree of its spatial organization (Clevers, 2013; Huch and Koo, 2015; Drost and Clevers, 2017). Similarly, the presence of midbrain-specific stem cell niches and clusters of mDANs was shown in a NSC-derived hMO model and approaches to efficiently differentiate mDANs within hMOs by starting from expandable neural precursor cells (NPCs) were reported (Monzel et al., 2017; Smits et al., 2019a, b; Tables 2, 3). In these approaches NPCs that received already some patterning toward the midbrain have been used. Both hMO models have been already used in other studies (Berger et al., 2018; Jan et al., 2018; Jarazo et al., 2019). Among the published hMO protocols, different approaches have been presented to estimate the number of mDANs that arise during the organoid development (Table 2). Tieng et al. (2014) dissociated their 3D structures and cultured the resulting single cells as a monolayer (Qian et al., 2016). Their quantification resulted in more than 60% TH-positive cells in 21 day-old cultures (Tieng et al., 2014) and around 55% TH-positive cells after 65 days (Qian et al., 2016). A more straightforward method to quantify the mDAN population is flow cytometry via fluorescence-activated cell sorting (FACS), which also requires dissociated hMOs. By doing so, it has been shown that after 61 days of culture around 64% cells were triple-positive for the mDAN markers TH, LMX1A, and FOXA2 (Monzel et al., 2017). Surprisingly, with the same method, Jo et al. (2016) identified only around 22% of MAP2-positive neurons co-expressing TH in their hMOs after 60 days of culture. A third approach to estimate the percentage of mDANs within hMOs has been described recently (Bolognin et al., 2019; Smits et al., 2019b). Here, image analysis algorithms enabled the automated segmentation of nuclei and neurons, with around 62% TH-positive cells after 35 days and around 54% TH-positive cells after 70 days of 3D culture were quantified. The application of high-content image-analysis allows entire organoid sections to be examined, rather than dissociated single cells, which preserves the original morphology and cell-cell interactions of the mDANs. TH is a marker commonly used for mDAN detection, as it is a rate-limiting enzyme for DA biosynthesis. However, it is also expressed in other catecholaminergic cell types and does not represent a unique marker specifically for mDANs, as in the case of the dopamine transporter (DAT) for example or the actual presence of the neurotransmitter DA (Abeliovich and Hammond, 2007). DAT expression has been reported in almost every hMO model (Jo et al., 2016; Qian et al., 2016; Monzel et al., 2017; Kim et al., 2019; Smits et al., 2019b; Table 2).

**TABLE 2:** Comparison of characteristics of different hMO derivation protocols: Overview of hMO-specific features reported in Tieng et al., 2014; Jo et al., 2016; Qian et al., 2016; Monzel et al., 2017; Kim et al., 2019; Smits et al., 2019b (*, different methods applied to determine and calculate TH content; °, different methods applied to determine DA content).

	**Tieng et al. (2014)**	**Qian et al. (2016)**	**Jo et al. (2016)**	**Monzel et al. (2017)**	**Kim et al. (2019)**	**Smits et al. (2019b)**
**mDANs**						
TH*	>60% (d21)	55% (d65)	22% (d60)	64% (d61)	n/a	54% (d70)
DAT	n/a	Yes	Yes	Yes	Yes	Yes
DA°	Yes	Yes	Yes	Yes	Yes	Yes
NM	n/a	n/a	Yes	Yes	n/a	Yes
**Glial cells**						
Oligodendrocytes	Yes	n/a	Yes	Yes	n/a	n/a
Astrocytes	Yes	Yes	Yes	Yes	n/a	n/a
**Neuronal subtypes**						
GABAergic	No	n/a	Yes	n/a	n/a	Yes
Glutamatergic	Yes	n/a	n/a	n/a	n/a	Yes
Serotonergic	No	n/a	n/a	n/a	n/a	Yes

**TABLE 3:** Comparison of NSC-derived hMO models: Overview of selected features of hMOs approaches published in Monzel et al., 2017; Smits et al., 2019b.

	**Monzel et al. (2017)**	**Smits et al. (2019b)**
**Culture conditions**		
Used cell type	smNPCs	mfNPCs
Number of cells	9,000	3,000
Embedding	Yes	No
Agitation	Yes	No
**mDANs**		
TH + cells*	∼64% (d61)	∼54% (d70)
Regionalization	Yes	No
A9/A10 specificity	Yes	Yes
DAT	Yes (d61)	Yes (d70)
D2/D3 receptor responsive	Yes	Yes
DA release	No	Yes
NM	Yes (>d149)	Yes (>d100)
**Other cell types**		
Oligodendrocytes	Yes (d61)	n/a
Astrocytes	Yes (d61)	n/a
Neuronal subtypes	n/a	Yes

Neuronal subtypes refers to the analysis whether neurons other than dopaminergic neuros are determined. As starting cell population, small molecule neural precursor cells (smNPCs) are used in Monzel et al., 2017, while Smits et al. (2019b) starts with midbrain floor plate neural progenitor cells (mfNPCs). *, different methods applied to determine and calculate TH content.

Tieng et al. (2014) did not show the presence of DAT in their engineered nervous tissues (ENTs), but they were able to prove the synthesis of DA via high-performance liquid chromatography (HPLC) after cell lysis. Also Jo et al. (2016) and Kim et al. (2019) also applied the same method to assess the DA content within the hMOs. To verify that the mDANs were not only able to produce DA, but actually able to release the neurotransmitter, the supernatant of the 3D cultures can be analyzed with an enzyme-linked immunosorbent assay (ELISA). SMITS et al. detected a DA release that increased as the hMOs matured (Smits et al., 2019b). An interesting observation was reported for the first time by JO et al., where they detected insoluble, dark-colored deposits in their hMOs after approximately 60 days (Jo et al., 2016). Using a Fontana-Masson staining they confirmed that these granules were neuromelanin (NM), which is a by-product of DA biosynthesis and accumulates postnatally in the *substantia nigra pars compacta* (SNc) of the human brain (Zecca et al., 2003; Pasca, 2018; Kim et al., 2019). Other hMO culture protocols have also stimulated the production of NM (Monzel et al., 2017; Smits et al., 2019b; Table 2). The presence of NM is a unique feature of the primate brain (Pasca, 2018). It is neither apparent in mice brains nor murine midbrain-specific organoids (Fedorow et al., 2005; Dawson et al., 2010; Jo et al., 2016; Marton and Paşca, 2016). Wild-type mDANs, derived and cultured in monolayer conditions, only produce NM after an artificial inductions of progerin expression, which is associated with premature aging (Miller et al., 2013; Sterneckert et al., 2014). Membrane-bound, dense pigmented NM was also detected in long-term cultures of homozygous DJ-1 mutant and idiopathic PD patient-derived mDANs (Burbulla et al., 2017). Currently, it is unknown whether NM has a protective or damaging effect on the cell survival, however, it is proven that NM-containing mDANs of the SNc are especially vulnerable during the course of PD (Zecca et al., 2003; Jo et al., 2016; Marton and Paşca, 2016). Therefore, NM containing hMOs have a great potential to be used for *in vitro* PD modeling, possibly revealing specific phenotypes that are not present in wild-type 2D cultures or murine models. Moreover, hMOs provide the basis for future studies about the role of NM in PD (Marton and Paşca, 2016; Michel et al., 2016). Since mDANs are essential to model the human midbrain, hMO research has so far focused mainly on this specific type of neurons. Nevertheless, detailed *in vivo* studies have described that the released DA diffuses into synaptic regions of glutamatergic and GABAergic synapses and directly affects other striatal cell types, including the neurons forming the island-/striosome GABA pathway, striatal cholinergic interneurons and the striatal GABA interneurons, all possessing DA receptors (Calabresi et al., 2014; Borroto-Escuela et al., 2018). Furthermore, *substantia nigra* (SN) dopaminergic neurons are directly controlled by GABAergic input (Tepper and Lee, 2007). Evidence from these studies suggest that the presence of other neuronal subtypes is important to be able to model multifactorial disease like PD. A first transcriptional characterization of hMO was performed by Jo et al. (2016), showing that aspects of prenatal midbrain gene expression profiles were found in the organoids in contrast to the conventional 2D-derived mDANs (The GTEx Consortium, 2015; Lin et al., 2016). For a further validation of the genetic expression profile during the course of hMO development they suggested conducting a single-cell transcriptome analysis, as it has been shown before for cerebral organoids (Camp et al., 2015; Kageyama et al., 2018). In a recent study, single-cell transcriptomic data from hMOs demonstrated that there is an increased expression of neuronal- and stem cell-specific genes in 35 day- compared to 70 day-old hMOs, whereas exclusively the gene-gene correlations between only neuron-specific genes increased considerably at day 70 (Smits et al., 2019a). This signifies an increasing commitment of cells toward the neuronal cellular fate during the course of organoid development and further supports the finding of a progressive maturation of post-mitotic neurons. The identification of these neuron-specific genes revealed that the genes up-regulated at the earlier time point are particularly relevant in the processes of neurogenesis, neuronal migration and differentiation [for example, early B-cell factor 3 (EBF3) (Garcia-Dominguez, 2003) and L1 cell adhesion molecule (L1CAM) (Patzke et al., 2016)], whereas the up-regulated genes at the later time point have been implicated *in vivo* in a modulatory contribution to neurite extension [for example, repulsive guidance molecule B (RGMB), Ma et al., 2011]. This indicates a higher commitment of the cells toward their intended fate and a progressive maturation of post-mitotic neurons within the hMOs. Since the presence of neuronal subtypes, glutamatergic and GABAergic neurons have been reported in hMOs before (Tieng et al., 2014; Jo et al., 2016), the residence of specific neuronal subtypes has been addressed with the high-resolution single-cell analysis (Smits et al., 2019a). The expression of genes typical for dopaminergic, glutamatergic, GABAergic, and serotonergic neurons have been investigated and their presence further confirmed by immunohistochemistry staining for the respective neurotransmitters. This allows dopaminergic, glutamatergic and GABAergic neurons and a few serotonergic neurons to be robustly detected within hMOs (Table 2). So far, detailed analysis of the neuronal subtype’s function and their interaction has not been addressed. However, with regards to the fact that mDANs physiologically synapse in the striatum and not in the midbrain *in vivo*, hMOs are potentially limited as an *in vitro* model in this case.

Besides the detailed characterization of the neuronal population, also the analysis of astroglia and oligodendrocyte differentiation is also crucial for accurately modeling the human midbrain. The presence of astrocytes is essential for the formation of synapses and regular neuronal activity (Chung et al., 2015). Astrocytes are defined later than neurons during development, and their immunoreactivity is only detectable in hMOs after 35 days of cultivation (Molofsky et al., 2012; Chaboub and Deneen, 2013; Monzel et al., 2017; Table 2). Fast information transmission between neurons depends on axonal myelination, which is achieved by oligodendrocytes in the CNS. In most stem cell-based differentiation protocols, the differentiation into oligodendrocytes is extremely inefficient (Jablonska et al., 2010; Bunk et al., 2016). However, differentiation into oligodendrocytes and detected myelination of non- dopaminergic neurons has been achieved in hMOs (Monzel et al., 2017). Some neurites in these hMOs were ensheathed by oligodendrocytes and even structures such as the nodes of Ranvier became apparent, which are of critical importance for electrochemical transmission of signals in axons (Faivre-Sarrailh and Devaux, 2013). The feature of unmyelinated or thinly myelinated neurons is particularly well described for SNc mDANs, and explains why only about 30% of βIII Tubulin (TUJ1) and myelin basic protein (MBP) overlapping cells have been quantified in the hMO system (Braak and Del Tredici, 2004; Orimo et al., 2011; Sulzer and Surmeier, 2013; Monzel et al., 2017). To allow for future applications and improve the impact of hMOs in pathophysiological and pharmacological studies, the electrical activity and functional maturity of the midbrain-specific 3D cultures have been assessed. The presence or rather the co-localization of the presynaptic marker SYNAPTOPHYSIN and the postsynaptic marker PSD95 indicated direct contact between a pre- and a postsynapse (Monzel et al., 2017). The exact morphology of hMO-derived synapses until now has not been addressed in detail, although other hPSC-derived brain organoids already reflect many aspects of human synapse formation and function (Wilson and Newell-Litwa, 2018). Whole-cell patch recordings have been performed with sliced hMOs sections (Jo et al., 2016) or with neurons obtained from hMOs (Smits et al., 2019b). This is an established, but invasive method that allow specific neuronal subtypes to be identified and analyzed, however, the continuous development of an individual organoid cannot yet be followed in such detail. Alternatively, non-invasive recordings of extra cellular field potentials can be achieved by a MEA systems and allow insights into physiological properties of *in vitro* cultures and chronological analysis (Luhmann et al., 2016). Tieng et al. (2014) detected spontaneous and evoked electrophysiological activities in their ENTs. Furthermore, in the study of Monzel et al. (2017) spikes occurred closely in time on multiple electrodes, which indicates neuronal network synchronicity, were detected. To specifically determine the activity of different neuronal receptors within the organoid, the response to chemical compounds can be examined. The functionality of DA receptors has been tested with the application of quinpirole, a specific D2/D3 receptor agonist. Importantly, mDANs express D2 autoreceptors. This has previously been used in several studies and shown to effectively suppress the firing in hMOs (Jo et al., 2016; Monzel et al., 2017; Smits et al., 2019a). Together with the reported DA production and release, this strongly suggests that TH-positive neurons, developed in hMOs, exhibit electrophysiological and biochemical qualities of mature mDANs and express functional, quinpirole-responsive, DA receptors. To further isolate and attribute the recorded signals to neuronal subtypes, inhibitory and excitatory communication was additionally blocked with specific drugs following an established experimental design by Illes et al. (2014). Drugs like, Gabazine an antagonist of GABA receptors which result in a disinhibition of target neurons of GABAergic neurons, as well as NMDA-receptor and AMPA/Kainate-receptor antagonists inhibiting glutamatergic excitatory communication were used (Illes et al., 2014). Together with the characteristic hallmarks of synapse formation, consisting of a direct contact between pre- and postsynapses and composing the prerequisite for electrophysiological and neuronal network functionality, these experiments confirmed functional GABAergic and glutamatergic neurons within the hMOs, in addition to the functional DA receptors present. While neurons do not exist in isolation in the CNS but rather form functional networks with other neurons and non-neuronal cells, it is important to expand our research of neurodegenerative diseases using 3D models that are able to recapitulate cell autonomous as well as non-cell autonomous aspects. Utilizing 3D cell culture models that comprise a variety of neuronal subtypes could lead to new insights into the selective vulnerability, which is observed in neurodegeneration. Evidence suggests that specific regulation of the excitability of mDANs by other neuronal subtypes in the midbrain might explain their selective vulnerability in PD (Korotkova et al., 2004; Calabresi et al., 2014; Calabresi and Di Filippo, 2015). This underlines the importance and the enormous potential for future disease models that utilize hMOs, as they contain functionally connected heterogeneous neuronal populations.

## Disease Modeling in Midbrain-Specific Organoids

Organoids, specifically modeling the human midbrain, hold great promises for studying the human brain development and for modeling the neurodegenerative disorder PD. PD is the second most common degenerative neurological disorder after AD and is defined by the selective loss of mDANs in the SNc of the human midbrain. Intriguingly, after decades of research on PD, the molecular mechanisms underlying the initiation and progression of the neurodegenerative disease, commonly occurring as idiopathic form, have not been entirely revealed and remain largely elusive (Roybon et al., 2004). Therefore, the establishment of region-specific brain organoids offers new possibilities to study neuronal diseases that are linked to a specific part of the human brain, such as PD. Neurodegenerative disorders, such as PD, are typically considered to be age-associated diseases (Sepe et al., 2016; Xu et al., 2016). However, there is accumulating evidence that PD has a strong neurodevelopmental component that probably defines the susceptibility to develop the disease (Garcia-Reitboeck et al., 2013; Le Grand et al., 2015; Schwamborn, 2018). This finding supports the importance of human brain development models to investigate the disease’s underlying mechanisms. A recent publication provides a proof-of-principle study where either patient-derived or genetically modified hMOs harboring the disease-associated G2019S mutation in the *LRRK2* gene show PD-relevant phenotypes including reduced number of mDANs (Smits et al., 2019b). By evaluating the number of links (branching) and nodes (dendrite bifurcation points) of the mDANs developed within the different hMO groups, a significant reduction of the dopaminergic network complexity in the patient-derived TH-positive neurons was identified which is also known to occur in PD patients’ brain (Bernheimer et al., 1973; Kordower et al., 2013; Smits et al., 2019b). Based on another published hMO protocol, Kim et al. (2019) also derived organoids carrying the *LRRK2*-G2019S mutation (Jo et al., 2016). In line with the findings of Smits et al. (2019b) and Kim et al. (2019) discovered that the mDANs within the *LRRK2*-G2019S organoids reveal a decreased neurite length in comparison to the mDANs within the control organoids. They further assessed an overall decreased expression level of mDANs-specific markers, such as TH, aromatic l-amino acid decarboxylase (AADC), and DAT in their engineered *LRRK2*-G2019S hMOs (Kim et al., 2019). They achieved a partial recovery of those gene’s expression levels after treating the *LRRK2*-G2019S mutant organoids with the *LRRK2* kinase inhibitor GSK2578215A. This *LRRK2* inhibition suggests a positive impact on mDAN cell death and additionally proves that this 3D cell culture system is susceptible to investigating therapeutic strategies against PD (Kim et al., 2019). Kim et al. (2019) also determined hMOs carrying the *LRRK2*-G2019S mutation exhibit an abnormal localization of α-synuclein that is phosphorylated at serine 129 (pS129). They claim that pS129-α-synuclein is aberrantly expressed in mutated hMOs and they detected a *LRRK2*-G2019S mutation-dependent increase of thioflavin T-positive deposits in TH-positive neurons over time, even though the overall α-synuclein expression did not appear to increase (Kim et al., 2019). Surprisingly, these detected thioflavin T-positive deposits appeared to be extracellular and were not clearly overlapping with the TH signal. Whether these findings are reproducible with another analytical method, still needs to be explored further. Nevertheless, the assessment of PD-associated pathologies, such as the synucleinopathies, in human-specific advanced cell culture models is crucial due to the inherent differences between human and mouse mDAN vulnerability and as existing murine transgenic models have not been efficient in developing an accurate representation of the underlying disease mechanisms (Byers et al., 2012; Burbulla et al., 2017; Hemmer et al., 2018; Koh et al., 2018). In the study of Smits et al. (2019b) a significant increase of FOXA2-positive progenitor cells in the patient-specific organoids was demonstrated. Since FOXA2 is required for the generation of mDANs, it is hypothesized that this might be a compensatory response to an impaired specification of mDANs promoted by the mutated *LRRK2* gene (Sasaki et al., 1997), additionally it might be a results of a decreased differentiation potential of the progenitor cells. Similar compensatory mechanisms have been described in PD before and might represent an attempt to counteract neurodevelopmental defects induced by PD-specific mutations (Blesa et al., 2017). While introducing also isogenic control hMOs, it was also confirmed that the introduction of the *LRRK2*-G2019S mutation caused deleterious effects on the complexity of mDANs within a healthy background. On the contrary, *LRRK2*-G2019S gene correction within a PD patient background is not sufficient to rescue this effect. As *LRRK2*-G2019S is not fully penetrant and the probability of developing PD individually varies among the carriers, it is suggested that its pathological function comprises of additional pathways (Smith et al., 2006; Goldwurm et al., 2007). In this context, a permissive genetic background, due to cumulative genetic variants, might mediate and either enhance or diminish the *LRRK2*-induced neurodegeneration (Bolognin et al., 2019). Remarkably, in the analysis of all studied features, the PD patient-derived lines cluster together, independently of the presence or absence of the mutation (Smits et al., 2019b). This indicates that the genetic background of the PD patients, regardless of gene editing, accounts for most of the differences between the studied cell lines and seems to be a major discriminating factor. Thus, not the *LRRK2*-G2019S mutation but rather the genetic background of the patients constitutes the strongest contribution to the phenotypes and supports the hypothesis that the genetic background of PD patients can influence the degeneration of mDANs (Bolognin et al., 2019). These findings show that 3D hMOs and the corresponding mDANs represent powerful new tools for *in vitro* disease modeling. Importantly, in PD patients in the midbrain mainly the SN is affected while a neighboring region, the ventral tegmental area, which also contains DNs is largely unaffected. Current hMO models so far have not addressed this different vulnerability sufficiently. However, this is a limitation that certainly will be investigated in the future.

By deriving further hMOs that carry defects in genes that are known to cause PD we could broaden the understanding of disease-related abnormalities and the context in which they arise (Benson and Huntley, 2019). The patient-specific nature of these models also opens promising avenues for future personalized medicine approaches (Bu et al., 2016; Hillje and Schwamborn, 2016; Smits et al., 2019b).

## Perspectives

The generation and characterization of midbrain-specific organoid protocols and thereby the provision of sophisticated *in vitro* models to study both neurodevelopmental processes and neurodegenerative diseases of the human midbrain have led to novel findings in the field of advanced 3D *in vitro* cell culture systems. Furthermore, the reported PD-relevant phenotypes in PD patient-derived hMOs have proved that these methods are a powerful tool for human-specific *in vitro* disease modeling of neurological disorders.

For such applications, an organoid model should be reproducible and stable for extended cultivation and manipulation. Even though the organoid technology is a powerful asset in the field of brain research, the hMO models show intrinsic disadvantages and limitations (Berger et al., 2018; Wang Y. et al., 2018). The lack of a natural body axis or supportive tissue prevents an organoid’s organization that is identical to the pattern of the *in vivo* human brain (Lancaster et al., 2013; Kelava and Lancaster, 2016a; Wang Y. et al., 2018). The identification of a specific brain regions as well as the reproducibility might be imperfect, nevertheless it is unlikely to create the exact culture conditions found in the human brain *in utero* (Trujillo and Muotri, 2018). A major limitation of the hMOs presented here, as well as other published brain organoid systems, is the absence of vasculature, which restricts the supply of oxygen and nutrition, especially in the inner part of the organoids (Kelava and Lancaster, 2016a; Wang Y. et al., 2018). It also might limit the growth of organoids beyond a certain size and increase the appearance of dead cells in the center of the organoids (Giandomenico and Lancaster, 2017; Monzel et al., 2017; Berger et al., 2018). The cell number, and consequently the cell density within the organoid seem to play an important role and might be a target for improvement. The choice of the surrogate matrix, the starting point and duration of the differentiation are further aspects that can influence the fidelity of the 3D culture. Recently, brain organoids were successfully transplanted into a mouse brain and murine blood vessels could be detected in the grafts (Mansour et al., 2018). Even though the transplanted organoid mimicked more precisely the *in vivo* brain anatomy, this method bears the disadvantage of xenocontamination (Trujillo and Muotri, 2018; Wang Y. et al., 2018). The absence of microglia, the resident innate immune cells of the CNS, is another major disadvantage for disease modeling, as they are actively involved in the development and maturation of neurons. In the case of cerebral organoids, an adaptation for a microglia-containing organoid model has been recently published (Ormel et al., 2018). Since some hMOs are derived from the neuroectoderm and microglia originate developmentally from the mesoderm, there is only the possibility to integrate externally derived microglia or their precursors to the developing hMO (Muffat et al., 2016; Abud et al., 2017; Haenseler et al., 2017; Trujillo and Muotri, 2018; Wang Y. et al., 2018).

Future development of cell culture technologies will further improve brain-specific organoid models and will support both the investigation of more complicated interactions in the human brain and the modeling of a larger range of neurological disorders (Di Lullo and Kriegstein, 2017; Wang Y. et al., 2018). Despite the current limitations, we can conclude that the hMO system presented here, along with other models, may be a first step toward a more human patient-specific, probably even personalized, era of advanced disease modeling and therapy development.

## Author Contributions

LS and JS jointly wrote and edited this review.

## Conflict of Interest

The authors declare that the research was conducted in the absence of any commercial or financial relationships that could be construed as a potential conflict of interest.

## References

[B1] Abe-Fukasawa N. Otsuka K. Aihara A. Itasaki N. Nishino T. (2018). Novel 3D liquid cell culture method for anchorage-independent cell growth, cell imaging and automated drug screening. *Sci. Rep.* 8:3627. 10.1038/s41598-018-21950-5 29483620 PMC5827526

[B2] Abeliovich A. Hammond R. (2007). Midbrain dopamine neuron differentiation: factors and fates. *Dev. Biol.* 304 447–454. 10.1016/j.ydbio.2007.01.032 17331494

[B3] Abud E. M. Ramirez R. N. Martinez E. S. Carson M. J. Poon W. W. Blurton-jones M. (2017). iPSC-Derived human microglia-like cells to study neurological diseases. *Neuron* 94 278–293.e9. 10.1016/j.neuron.2017.03.042 28426964 PMC5482419

[B4] Arlotta P. (2018). Organoids required! A new path to understanding human brain development and disease. *Nat. Methods* 15 27–29. 10.1038/nmeth.4557 29298289

[B5] Benson D. L. Huntley G. W. (2019). Are we listening to everything the PARK genes are telling us? *J. Comp. Neurol.* 527 1527–1540. 10.1002/cne.24642 30680728 PMC6800668

[B6] Berger E. Magliaro C. Paczia N. Monzel A. S. Antony P. Linster C. L. (2018). Millifluidic culture improves human midbrain organoid vitality and differentiation. *Lab. Chip* 18 3172–3183. 10.1039/c8lc00206a 30204191

[B7] Bernheimer H. Birkmayer W. Hornykiewicz O. Jellinger K. Seitelberger F. (1973). Brain dopamine and the syndromes of Parkinson and huntington clinical, morphological and neurochemical correlations. *J. Neurol. Sci.* 20 415–455. 10.1016/0022-510X(73)90175-5 4272516

[B8] Bian S. Repic M. Guo Z. Kavirayani A. Burkard T. Bagley J. A. (2018). Genetically engineered cerebral organoids model brain tumor formation. *Nat. Methods* 15 631–639. 10.1038/s41592-018-0070-7 30038414 PMC6071863

[B9] Blesa J. Trigo-Damas I. Dileone M. del Rey N. L. G. Hernandez L. F. Obeso J. A. (2017). Compensatory mechanisms in Parkinson’s disease: circuits adaptations and role in disease modification. *Exp. Neurol.* 298 148–161. 10.1016/j.expneurol.2017.10.002 28987461

[B10] Bolognin S. Fossépré M. Qing X. Jarazo J. Ščančar J. Moreno E. L. (2019). 3D cultures of Parkinson’s disease-specific dopaminergic neurons for high content phenotyping and drug testing. *Adv. Sci.* 6:1800927 10.1002/advs.201800927 PMC6325628 30643711

[B11] Borroto-Escuela D. O. Perez De La Mora M. Manger P. Narváez M. Beggiato S. Crespo-Ramírez M. (2018). Brain dopamine transmission in health and Parkinson’s disease: modulation of synaptic transmission and plasticity through volume transmission and dopamine heteroreceptors. *Front. Synaptic Neurosci.* 10:20 10.3389/fnsyn.2018.00020 PMC6048293 30042672

[B12] Braak H. Del Tredici K. (2004). Poor and protracted myelination as a contributory factor to neurodegenerative disorders. *Neurobiol. Aging* 25 19–23. 10.1016/j.neurobiolaging.2003.04.001 14675725

[B13] Bu L.-L. Yang K. Xiong W.-X. Liu F.-T. Anderson B. Wang Y. (2016). Toward precision medicine in Parkinson’s disease. *Ann. Transl. Med.* 4:26 10.3978/j.issn.2305-5839.2016.01.21 PMC4731609 26889479

[B14] Bunk E. C. Ertaylan G. Ortega F. Pavlou M. A. Gonzalez Cano L. Stergiopoulos A. (2016). Prox1 is required for oligodendrocyte cell identity in adult neural stem cells of the subventricular zone. *Stem Cells* 34 2115–2129. 10.1002/stem.2374 27068685

[B15] Burbulla L. F. Song P. Mazzulli J. R. Zampese E. Wong Y. C. Jeon S. (2017). Dopamine oxidation mediates mitochondrial and lysosomal dysfunction in Parkinson’s disease. *Science* 357 1255–1261. 10.1126/science.aam9080 28882997 PMC6021018

[B16] Byers B. Lee H. Reijo Pera R. (2012). Modeling Parkinson’s disease using induced pluripotent stem cells. *Curr. Neurol. Neurosci. Rep.* 12 237–242. 10.1007/s11910-012-0270-y 22538490 PMC3342500

[B17] Calabresi P. Di Filippo M. (2015). The changing tree in Parkinson’s disease. *Nat. Neurosci.* 18 1196–1198. 10.1038/nn.4092 26308978

[B18] Calabresi P. Picconi B. Tozzi A. Ghiglieri V. Di Filippo M. (2014). Direct and indirect pathways of basal ganglia: a critical reappraisal. *Nat. Neurosci.* 17 1022–1030. 10.1038/nn.3743 25065439

[B19] Camp J. G. Badsha F. Florio M. Kanton S. Gerber T. Wilsch-Bräuninger M. (2015). Human cerebral organoids recapitulate gene expression programs of fetal neocortex development. *Proc. Natl. Acad. Sci. U.S.A.* 112 15672–15677. 10.1073/pnas.1520760112 26644564 PMC4697386

[B20] Chaboub L. S. Deneen B. (2013). Astrocyte form and function in the developing central nervous system. *Semin. Pediatr. Neurol.* 20 230–235. 10.1016/J.SPEN.2013.10.003 24365570 PMC3874818

[B21] Chambers S. M. Fasano C. A. Papapetrou E. P. Tomishima M. Sadelain M. Studer L. (2009). Highly efficient neural conversion of human ES and iPS cells by dual inhibition of SMAD signaling. *Nat. Biotechnol.* 27 275–280. 10.1038/nbt.1529 19252484 PMC2756723

[B22] Choi S. H. Kim Y. H. Hebisch M. Sliwinski C. Lee S. D’Avanzo C. (2014). A three-dimensional human neural cell culture model of Alzheimer’s disease. *Nature* 515 274–278. 10.1038/nature13800 25307057 PMC4366007

[B23] Choi S. H. Kim Y. H. Quinti L. Tanzi R. E. Kim D. Y. (2016). 3D culture models of Alzheimer’s disease: a road map to a “cure-in-a-dish.”. *Mol. Neurodegener.* 11:75. 10.1186/s13024-016-0139-7 27938410 PMC5148918

[B24] Chung W.-S. Welsh C. A. Barres B. A. Stevens B. (2015). Do glia drive synaptic and cognitive impairment in disease? *Nat. Neurosci.* 18 1539–1545. 10.1038/nn.4142 26505565 PMC4739631

[B25] Clevers H. (2013). The intestinal crypt, a prototype stem cell compartment. *Cell* 154 274–284. 10.1016/j.cell.2013.07.004 23870119

[B26] Clevers H. (2016). Modeling development and disease with organoids. *Cell* 165 1586–1597. 10.1016/j.cell.2016.05.082 27315476

[B27] Cooper O. Seo H. Andrabi S. Guardia-Laguarta C. Graziotto J. Sundberg M. (2012). Pharmacological rescue of mitochondrial deficits in iPSC-derived neural cells from patients with familial Parkinson’s disease. *Sci. Transl. Med.* 4:141ra90 10.1126/scitranslmed.3003985 PMC3462009 22764206

[B28] Cugola F. R. Fernandes I. R. Russo F. B. Freitas B. C. Dias J. L. M. Guimarães K. P. (2016). The Brazilian Zika virus strain causes birth defects in experimental models. *Nature* 534 267–271. 10.1038/nature18296 27279226 PMC4902174

[B29] Cullen D. K. Wolf J. A. Vernekar V. N. Vukasinovic J. LaPlaca M. C. (2012). Neural tissue engineering and biohybridized microsystems for neurobiological investigation in vitro (Part 1). *Crit. Rev. Biomed. Eng.* 39 201–240. 10.1615/critrevbiomedeng.v39.i3.30 21967303

[B30] Dang J. Tiwari S. K. Lichinchi G. Qin Y. Patil V. S. Eroshkin A. M. (2016). Zika Virus depletes neural progenitors in human cerebral organoids through activation of the innate immune receptor TLR3. *Stem Cell* 19 258–265. 10.1016/j.stem.2016.04.014 27162029 PMC5116380

[B31] D’Avanzo C. Aronson J. Kim Y. H. Choi S. H. Tanzi R. E. Kim D. Y. (2015). Alzheimer’s in 3D culture: challenges and perspectives. *Bioessays* 37 1139–1148. 10.1002/bies.201500063 26252541 PMC4674791

[B32] Dawson T. M. Ko H. S. Dawson V. L. (2010). Genetic animal models of Parkinson’s disease. *Neuron* 66 646–661. 10.1016/j.neuron.2010.04.034 20547124 PMC2917798

[B33] Di Lullo E. Kriegstein A. R. (2017). The use of brain organoids to investigate neural development and disease. *Nat. Rev. Neurosci.* 18 573–584. 10.1038/nrn.2017.107 28878372 PMC5667942

[B34] Doi D. Samata B. Katsukawa M. Kikuchi T. Morizane A. Ono Y. (2014). Isolation of human induced pluripotent stem cell-derived dopaminergic progenitors by cell sorting for successful transplantation. *Stem Cell Rep.* 2 337–350. 10.1016/j.stemcr.2014.01.013 24672756 PMC3964289

[B35] Drost J. Clevers H. (2017). Translational applications of adult stem cell-derived organoids. *Company Biol.* 144 968–975. 10.1242/dev.140566 28292843

[B36] Dutta D. Heo I. Clevers H. (2017). Disease modeling in stem cell-derived 3D organoid systems. *Trends Mol. Med.* 23 393–410. 10.1016/j.molmed.2017.02.007 28341301

[B37] Faivre-Sarrailh C. Devaux J. J. (2013). Neuro-glial interactions at the nodes of Ranvier: implication in health and diseases. *Front. Cell. Neurosci.* 7:196. 10.3389/fncel.2013.00196 24194699 PMC3810605

[B38] Fatehullah A. Tan S. H. Barker N. (2016). Organoids as an in vitro model of human development and disease. *Nat. Publishing Group* 18 246–254. 10.1038/ncb3312 26911908

[B39] Fedorow H. Tribl F. Halliday G. Gerlach M. Riederer P. Double K. L. (2005). Neuromelanin in human dopamine neurons: comparison with peripheral melanins and relevance to Parkinson’s disease. *Prog. Neurobiol.* 75 109–124. 10.1016/j.pneurobio.2005.02.001 15784302

[B40] Garcez P. P. Loiola E. C. Da Costa R. M. Higa L. M. Trindade P. Delvecchio R. (2016). Zika virus impairs growth in human neurospheres and brain organoids. *Science* 352 816–818. 10.1126/science.aaf6116 27064148

[B41] Garcia-Dominguez M. (2003). Ebf gene function is required for coupling neuronal differentiation and cell cycle exit. *Development* 130 6013–6025. 10.1242/dev.00840 14573522

[B42] Garcia-Reitboeck P. Anichtchik O. Dalley J. W. Ninkina N. Tofaris G. K. Buchman V. L. (2013). Endogenous alpha-synuclein influences the number of dopaminergic neurons in mouse substantia nigra. *Exp. Neurol.* 248 541–545. 10.1016/J.EXPNEUROL.2013.07.015 23933574 PMC4104299

[B43] Giandomenico S. L. Lancaster M. A. (2017). Probing human brain evolution and development in organoids. *Curr. Opin. Cell Biol.* 44 36–43. 10.1016/j.ceb.2017.01.001 28157638

[B44] Goldwurm S. Zini M. Mariani L. Tesei S. Miceli R. Sironi F. (2007). Evaluation of LRRK2 G2019S penetrance: relevance for genetic counseling in Parkinson disease. *Neurology* 68 1141–1143. 10.1212/01.wnl.0000254483.19854.ef 17215492

[B45] Grealish S. Diguet E. Parmar M. Bjo A. Grealish S. Diguet E. (2014). Human ESC-Derived dopamine neurons show similar preclinical efficacy and potency to fetal neurons when grafted in a rat model of Parkinson’s clinical progress human ESC-derived dopamine neurons show similar preclinical efficacy and potency to fetal neur. *Cell Stem Cell* 15 653–665. 10.1016/j.stem.2014.09.017 25517469 PMC4232736

[B46] Haenseler W. Zambon F. Lee H. Vowles J. Rinaldi F. Duggal G. (2017). Excess α-synuclein compromises phagocytosis in iPSC-derived macrophages. *Sci. Rep.* 7:9003. 10.1038/s41598-017-09362-3 28827786 PMC5567139

[B47] Hargus G. Cooper O. Deleidi M. Levy A. Lee K. Marlow E. (2010). Differentiated Parkinson patient-derived induced pluripotent stem cells grow in the adult rodent brain and reduce motor asymmetry in Parkinsonian rats. *Proc. Natl. Acad. Sci. U.S.A.* 107 15921–15926. 10.1073/pnas.1010209107 20798034 PMC2936617

[B48] Haycock J. W. (2011). 3D cell culture: a review of current approaches and techniques. *Methods Mol. Biol. Biol.* 695 1–5. 10.1007/978-1-60761-984-0_1 21042962

[B49] Hemmer K. Smits L. M. Bolognin S. Schwamborn J. C. (2018). In vivo phenotyping of human Parkinson’s disease-specific stem cells carrying the LRRK2-G2019S mutation reveals increased a-Synuclein levels but absence of spreading. *Opera Med. Physiol.* 4 71–77. 10.20388/omp2018.001.0057

[B50] Hillje A.-L. Schwamborn J. C. (2016). Utilization of stem cells to model Parkinson’s disease – current state and future challenges. *Future Neurol.* 11 171–186. 10.2217/fnl.16.7

[B51] Hubert C. G. Rivera M. Spangler L. C. Wu Q. Mack S. C. Prager B. C. (2016). A three-dimensional organoid culture system derived from human glioblastomas recapitulates the hypoxic gradients and cancer stem cell heterogeneity of tumors found in vivo. *Cancer Res.* 76 2465–2477. 10.1158/0008-5472.can-15-2402 26896279 PMC4873351

[B52] Huch M. Koo B. (2015). Modeling mouse and human development using organoid cultures. *Development* 142 3113–3125. 10.1242/dev.118570 26395140

[B53] Illes S. Jakab M. Beyer F. Gelfert R. Couillard-Despres S. Schnitzler A. (2014). Intrinsically active and pacemaker neurons in pluripotent stem cell-derived neuronal populations. *Stem Cell Rep.* 2 323–336. 10.1016/j.stemcr.2014.01.006 24672755 PMC3964285

[B54] Jablonska B. Aguirre A. Raymond M. Szabo G. Kitabatake Y. Sailor K. A. (2010). Chordin-induced lineage plasticity of adult SVZ neuroblasts after demyelination. *Nat. Neurosci.* 13 541–550. 10.1038/nn.2536 20418875 PMC4059417

[B55] Jan A. Jansonius B. Delaidelli A. Bhanshali F. An Y. A. Ferreira N. (2018). Activity of translation regulator eukaryotic elongation factor-2 kinase is increased in Parkinson disease brain and its inhibition reduces alpha synuclein toxicity. *Acta Neuropathol. Commun.* 6:54. 10.1186/s40478-018-0554-9 29961428 PMC6027557

[B56] Jarazo J. Barmpa K. Rosety I. Smits L. M. Arias-Fuenzalida J. Walter J. (2019). Parkinson’s disease phenotypes in patient specific brain organoids are improved by HP-β-CD treatment. *BioRxiv* [Preprint] 10.1101/813089

[B57] Jo J. Xiao Y. Xuyang Sun A. King Tan E. Shawn Je H. Ng H. (2016). Midbrain-like organoids from human pluripotent stem cells contain functional dopaminergic and neuromelanin-producing neurons. *Cell Stem Cell* 19 248–257. 10.1016/j.stem.2016.07.005 27476966 PMC5510242

[B58] Kadoshima T. Sakaguchi H. Nakano T. Soen M. Ando S. Eiraku M. (2013). Self-organization of axial polarity, inside-out layer pattern, and species-specific progenitor dynamics in human ES cell-derived neocortex. *Proc. Natl. Acad. Sci. U.S.A.* 110 20284–20289. 10.1073/pnas.1315710110 24277810 PMC3864329

[B59] Kageyama J. Wollny D. Treutlein B. Camp J. G. (2018). ShinyCortex: exploring single-cell transcriptome data from the developing human cortex. *Front. Neurosci.* 12:315. 10.3389/fnins.2018.00315 29867326 PMC5962798

[B60] Kelava I. Lancaster M. A. (2016a). Dishing out mini-brains: current progress and future prospects in brain organoid research. *Dev. Biol.* 420 199–209. 10.1016/j.ydbio.2016.06.037 27402594 PMC5161139

[B61] Kelava I. Lancaster M. A. (2016b). Stem cell models of human brain development. *Cell Stem Cell* 18 736–748. 10.1016/j.stem.2016.05.022 27257762

[B62] Kim H. Park H. J. Choi H. Chang Y. Park H. Shin J. (2019). Modeling G2019S-LRRK2 sporadic Parkinson’s disease in 3D midbrain organoids. *Stem Cell Rep.* 12 518–531. 10.1016/J.STEMCR.2019.01.020 30799274 PMC6410341

[B63] Kirkeby A. Grealish S. Wolf D. A. Nelander J. Wood J. Lundblad M. (2012). Generation of regionally specified neural progenitors and functional neurons from human embryonic stem cells under defined conditions. *Cell Rep.* 1 703–714. 10.1016/j.celrep.2012.04.009 22813745

[B64] Koh Y. H. Tan L. Y. Ng S. (2018). Patient-Derived induced pluripotent stem cells and organoids for modeling alpha synuclein propagation in Parkinson’s disease. *Front. Cell. Neurosci.* 12:413 10.3389/fncel.2018.00413 PMC6240766 30483063

[B65] Kordower J. H. Olanow C. W. Dodiya H. B. Chu Y. Beach T. G. Adler C. H. (2013). Disease duration and the integrity of the nigrostriatal system in Parkinson’s disease. *Brain* 136 2419–2431. 10.1093/brain/awt192 23884810 PMC3722357

[B66] Korotkova T. M. Ponomarenko A. A. Brown R. E. Haas H. L. (2004). Functional diversity of ventral midbrain dopamine and GABAergic neurons. *Mol. Neurobiol.* 29 243–259. 10.1385/MN:29:3:243 15181237

[B67] Kriks S. Shim J.-W. Piao J. Ganat Y. M. Wakeman D. R. Xie Z. (2011). Dopamine neurons derived from human ES cells efficiently engraft in animal models of Parkinson’s disease. *Nature* 480 547–551. 10.1038/nature10648 22056989 PMC3245796

[B68] Lancaster M. A. Corsini N. S. Burkard T. R. Knoblich J. A. (2016). Guided self-organization recapitulates tissue architecture in a bioengineered brain organoid model. *BioRxiv* [Preprint] 10.1101/049346

[B69] Lancaster M. A. Knoblich J. A. (2014a). Generation of cerebral organoids from human pluripotent stem cells. *Nat. Protoc.* 9:2329 10.1038/nprot.2014.158 PMC4160653 25188634

[B70] Lancaster M. A. Knoblich J. A. (2014b). Organogenesis in a dish: modeling development and disease using organoid technologies. *Science* 345:1247125. 10.1126/science.1247125 25035496

[B71] Lancaster M. A. Renner M. Martin C.-A. Wenzel D. Bicknell L. S. Hurles M. E. (2013). Cerebral organoids model human brain development and microcephaly. *Nature* 501 373–379. 10.1038/nature12517 23995685 PMC3817409

[B72] Le Grand J. N. Gonzalez-Cano L. Pavlou M. A. Schwamborn J. C. (2015). Neural stem cells in Parkinson’s disease: a role for neurogenesis defects in onset and progression. *Cell. Mol. Life Sci.* 72 773–797. 10.1007/s00018-014-1774-1 25403878 PMC11113294

[B73] Lin L. Göke J. Cukuroglu E. Dranias M. R. VanDongen A. M. J. Stanton L. W. (2016). Molecular features underlying neurodegeneration identified through in vitro modeling of genetically diverse Parkinson’s disease patients. *Cell Rep.* 15 2411–2426. 10.1016/j.celrep.2016.05.022 27264186

[B74] Luhmann H. J. Sinning A. Yang J. Reyes-puerta V. (2016). Spontaneous neuronal activity in developing neocortical networks: from single cells to large-scale interactions. *Front. Neural Circ.* 10:40. 10.3389/fncir.2016.00040 27252626 PMC4877528

[B75] Ma C. H. Brenner G. J. Omura T. Samad O. A. Costigan M. Inquimbert P. (2011). The BMP coreceptor RGMb promotes while the endogenous BMP antagonist noggin reduces neurite outgrowth and peripheral nerve regeneration by modulating BMP signaling. *J Neurosci.* 31 18391–18400. 10.1523/JNEUROSCI.4550-11.2011 22171041 PMC3243947

[B76] Mansour A. A. Gonçalves J. T. Bloyd C. W. Li H. Fernandes S. Quang D. (2018). An *in vivo* model of functional and vascularized human brain organoids. *Nat. Biotechnol.* 36 432–441. 10.1038/nbt.4127 29658944 PMC6331203

[B77] Marton R. M. Paşca S. P. (2016). Neural differentiation in the third dimension: generating a human midbrain. *Cell Stem Cell* 19 145–146. 10.1016/j.stem.2016.07.017 27494668

[B78] Method of the Year 2017: Organoids. (2018). Method of the year 2017: organoids. *Nat. Methods* 15:1 10.1038/nmeth.4575

[B79] Michel P. P. Hirsch E. C. Hunot S. (2016). Understanding dopaminergic cell death pathways in Parkinson disease. *Neuron* 90 675–691. 10.1016/j.neuron.2016.03.038 27196972

[B80] Miller J. D. Ganat Y. M. Kishinevsky S. Bowman R. L. Liu B. Tu E. Y. (2013). Human iPSC-based modeling of late-onset disease via progerin-induced aging. *Cell Stem Cell* 13 691–705. 10.1016/j.stem.2013.11.006 24315443 PMC4153390

[B81] Miner J. J. Diamond M. S. (2016). Understanding how zika virus enters and infects neural target cells. *Cell Stem Cell* 18 559–560. 10.1016/j.stem.2016.04.009 27152436

[B82] Molofsky A. V. Krencik R. Krenick R. Ullian E. M. Ullian E. Tsai H. (2012). Astrocytes and disease: a neurodevelopmental perspective. *Genes Dev.* 26 891–907. 10.1101/gad.188326.112 22549954 PMC3347787

[B83] Monzel A. S. Smits L. M. Hemmer K. Hachi S. Moreno E. L. van Wuellen T. (2017). Derivation of human midbrain-specific organoids from neuroepithelial stem cells. *Stem Cell Rep.* 8 1–11. 10.1016/j.stemcr.2017.03.010 28416282 PMC5425618

[B84] Muffat J. Li Y. Yuan B. Mitalipova M. Omer A. Corcoran S. (2016). Efficient derivation of microglia-like cells from human pluripotent stem cells. *Nat. Med.* 22 1358–1367. 10.1038/nm.4189 27668937 PMC5101156

[B85] Nguyen H. N. Byers B. Cord B. Shcheglovitov A. Byrne J. Gujar P. (2011). LRRK2 mutant iPSC-derived DA neurons demonstrate increased susceptibility to oxidative stress. *Cell Stem Cell* 8 267–280. 10.1016/j.stem.2011.01.013 21362567 PMC3578553

[B86] Nolbrant S. Heuer A. Parmar M. Kirkeby A. (2017). Generation of high-purity human ventral midbrain dopaminergic progenitors for in vitro maturation and intracerebral transplantation. *Nat. Protoc.* 12 1962–1979. 10.1038/nprot.2017.078 28858290

[B87] Nowakowski T. J. Pollen A. A. Di Lullo E. Sandoval-Espinosa C. Bershteyn M. Kriegstein A. R. (2016). Expression analysis highlights AXL as a candidate zika virus entry receptor in neural stem cells. *Cell Stem Cell* 18 591–596. 10.1016/j.stem.2016.03.012 27038591 PMC4860115

[B88] Orimo S. Uchihara T. Kanazawa T. Itoh Y. Wakabayashi K. Kakita A. (2011). Unmyelinated axons are more vulnerable to degeneration than myelinated axons of the cardiac nerve in Parkinson’s disease. *Neuropathol. Appl. Neurobiol.* 37 791–802. 10.1111/j.1365-2990.2011.01194.x 21696416

[B89] Ormel P. R. Vieira de Sa R. Hol E. M. Pasterkamp R. J. Vieira de Sá R. van Bodegraven E. J. (2018). Microglia innately develop within cerebral organoids. *Nat. Commun.* 9:4167. 10.1038/s41467-018-06684-2 30301888 PMC6177485

[B90] Paşca A. M. Sloan S. A. Clarke L. E. Tian Y. Makinson C. D. Huber N. (2015). Functional cortical neurons and astrocytes from human pluripotent stem cells in 3D culture. *Nat. Methods* 12 671–678. 10.1038/nmeth.3415 26005811 PMC4489980

[B91] Pasca S. P. (2018). The rise of three-dimensional human brain cultures. *Nat. Rev.* 553 437–445. 10.1038/nature25032 29364288

[B92] Patzke C. Acuna C. Giam L. R. Wernig M. Südhof T. C. (2016). Conditional deletion of L1CAM in human neurons impairs both axonal and dendritic arborization and action potential generation. *J. Exp. Med.* 213 499–515. 10.1084/jem.20150951 27001749 PMC4821644

[B93] Qian X. Nguyen H. N. Song M. M. Hadiono C. Ogden S. C. Hammack C. (2016). Brain-Region-Specific organoids using mini-bioreactors for modeling ZIKV exposure. *Cell* 165 1238–1254. 10.1016/j.cell.2016.04.032 27118425 PMC4900885

[B94] Qing X. Walter J. Jarazo J. Arias-Fuenzalida J. Hillje A. L. Schwamborn J. C. (2017). CRISPR/Cas9 and piggyBac-mediated footprint-free LRRK2-G2019S knock-in reveals neuronal complexity phenotypes and α-Synuclein modulation in dopaminergic neurons. *Stem Cell Res.* 24 44–50. 10.1016/j.scr.2017.08.013 28826027

[B95] Raja W. K. Mungenast A. E. Lin Y. Ko T. Abdurrob F. Seo J. (2016). Self-Organizing 3D human neural tissue derived from induced pluripotent stem cells recapitulate Alzheimer’s disease phenotypes. *PLoS One* 11:e0161969 10.1371/journal.pone.0161969 PMC5021368 27622770

[B96] Reinhardt P. Glatza M. Hemmer K. Tsytsyura Y. Thiel C. S. Hoing S. (2013a). Derivation and expansion using only small molecules of human neural progenitors for neurodegenerative disease modeling. *PLoS One* 8:e59252. 10.1371/journal.pone.0059252 23533608 PMC3606479

[B97] Reinhardt P. Schmid B. Burbulla L. F. Schöndorf D. C. Wagner L. Glatza M. (2013b). Genetic correction of a lrrk2 mutation in human iPSCs links parkinsonian neurodegeneration to ERK-dependent changes in gene expression. *Cell Stem Cell* 12 354–367. 10.1016/j.stem.2013.01.008 23472874

[B98] Renner M. Lancaster M. A. Bian S. Choi H. Ku T. Peer A. (2017). Self-organized developmental patterning and differentiation in cerebral organoids. *EMBO J.* 36 1316–1329. 10.15252/embj.201694700 28283582 PMC5430225

[B99] Roybon L. Christophersen N. S. Brundin P. Li J.-Y. (2004). Stem cell therapy for Parkinson’s disease: where do we stand? *Cell Tissue Res.* 318 261–273. 10.1007/s00441-004-0946-y 15309619

[B100] Ryan S. D. Dolatabadi N. Chan S. F. Zhang X. Akhtar M. W. Parker J. (2013). Isogenic human iPSC Parkinson’s model shows nitrosative stress-induced dysfunction in MEF2-PGC1α transcription. *Cell* 155 1351–1364. 10.1016/j.cell.2013.11.009 24290359 PMC4028128

[B101] Sánchez-Danés A. Richaud-Patin Y. Carballo-Carbajal I. Jiménez-Delgado S. Caig C. Mora S. (2012). Disease-specific phenotypes in dopamine neurons from human iPS-based models of genetic and sporadic Parkinson’s disease. *EMBO Mol. Med.* 4 380–395. 10.1002/emmm.201200215 22407749 PMC3403296

[B102] Sasaki H. Hui C. Nakafuku M. Kondoh H. (1997). A binding site for Gli proteins is essential for HNF-3beta floor plate enhancer activity in transgenics and can respond to Shh in vitro. *Development* 124 1313–1322. 9118802 10.1242/dev.124.7.1313

[B103] Sato T. Vries R. G. Snippert H. J. van de Wetering M. Barker N. Stange D. E. (2009). Single Lgr5 stem cells build cryptvillus structures in vitro without a mesenchymal niche. *Nature* 459 262–265. 10.1038/nature07935 19329995

[B104] Schwamborn J. C. (2018). Is Parkinson’s disease a neurodevelopmental disorder and will brain organoids help us to understand it? *Stem Cells Dev.* 27 968–975. 10.1089/scd.2017.0289 29415619

[B105] Seidel D. Krinke D. Jahnke H. G. Hirche A. Kloß D. Mack T. G. A. (2012). Induced tauopathy in a novel 3D-Culture model mediates neurodegenerative processes: a real-time study on biochips. *PLoS One* 7:e49150. 10.1371/journal.pone.0049150 23145103 PMC3492324

[B106] Sepe S. Milanese C. Gabriels S. Derks K. W. J. Payan-Gomez C. van IJcken W. F. J. (2016). Inefficient DNA repair is an aging-related modifier of parkinson’s disease. *Cell Rep.* 15 1866–1875. 10.1016/j.celrep.2016.04.071 27210754 PMC4893155

[B107] Setia H. Muotri A. R. (2019). Brain organoids as a model system for human neurodevelopment and disease. *Semin. Cell Dev. Biol.* 95 93–97. 10.1016/j.semcdb.2019.03.002 30904636 PMC6755075

[B108] Smith W. W. Pei Z. Jiang H. Dawson V. L. Dawson T. M. Ross C. A. (2006). Kinase activity of mutant LRRK2 mediates neuronal toxicity. *Nat. Neurosci.* 9 1231–1233. 10.1038/nn1776 16980962

[B109] Smits L. M. Magni S. Grzyb K. Antony P. M. Krüger R. Skupin A. (2019a). Single-cell transcriptomics reveals multiple neuronal cell types in human midbrain-specific organoids. *BioRxiv* [Preprint] 10.1101/589598 PMC7683480 32737576

[B110] Smits L. M. Reinhardt L. Reinhardt P. Glatza M. Monzel A. S. Stanslowsky N. (2019b). Modeling Parkinson’s disease in midbrain-like organoids. *NPJ Parkinson Dis.* 5:5. 10.1038/s41531-019-0078-4 30963107 PMC6450999

[B111] Spathis A. D. Asvos X. Ziavra D. Karampelas T. Topouzis S. Cournia Z. (2017). Nurr1:RXRα heterodimer activation as monotherapy for Parkinson’ s disease. *Proc. Natl. Acad. Sci. U.S.A.* 114 3999–4004. 10.1073/pnas.1616874114 28348207 PMC5393203

[B112] Sterneckert J. L. Reinhardt P. Schöler H. R. (2014). Investigating human disease using stem cell models. *Nat. Rev. Genet.* 15 625–639. 10.1038/nrg3764 25069490

[B113] Sulzer D. Surmeier D. J. (2013). Neuronal vulnerability, pathogenesis, and Parkinson’s disease. *Mov. Disord.* 28 41–50. 10.1002/mds.25095 22791686 PMC3578396

[B114] Tepper J. M. Lee C. R. (2007). GABAergic control of substantia nigra dopaminergic neurons. *Prog. Brain Res.* 160 189–208. 10.1016/S0079-6123(06)60011-3 17499115

[B115] The GTEx Consortium (2015). The genotype-tissue expression (GTEx) pilot analysis: multitissue gene regulation in humans. *Science* 348 648–660. 10.1126/science.1262110 25954001 PMC4547484

[B116] Tieng V. Stoppini L. Villy S. Fathi M. Dubois-Dauphin M. Krause K.-H. (2014). Engineering of midbrain organoids containing long-lived dopaminergic neurons. *Stem Cells Dev.* 23 1–32. 10.1089/scd.2013.0442 24576173

[B117] Trujillo C. A. Muotri A. R. (2018). Brain organoids and the study of neurodevelopment. *Trends Mol. Med.* 24 982–990. 10.1016/j.molmed.2018.09.005 30377071 PMC6289846

[B118] Wang X. Allen W. E. Wright M. A. Sylwestrak E. L. Samusik N. Vesuna S. (2018). Three-dimensional intact-tissue sequencing of single-cell transcriptional states. *Science* 361 1–9. 10.1126/science.aat5691 29930089 PMC6339868

[B119] Wang Y. Zhu Y. Qin J. (2018). Human brain organoid-on-a-chip to model prenatal nicotine exposure. *Lab. Chip* 18 851–860. 10.1039/C7LC01084B 29437173

[B120] Wells M. F. Salick M. R. Wiskow O. Ho D. J. Worringer K. A. Ihry R. J. (2016). Genetic ablation of AXL does not protect human neural progenitor cells and cerebral organoids from zika virus infection. *Cell Stem Cell* 19 703–708. 10.1016/j.stem.2016.11.011 27912091

[B121] Wilson E. S. Newell-Litwa K. (2018). Stem cell models of human synapse development and degeneration. *Mol. Biol. Cell* 29 2913–2921. 10.1091/mbc.E18-04-0222 30475098 PMC6329912

[B122] Xu Y. Yang J. Shang H. (2016). Meta-analysis of risk factors for Parkinson’s disease dementia. *Transl. Neurodegener.* 5:11 10.1186/s40035-016-0058-0 PMC4890279 27257478

[B123] Zecca L. Zucca F. A. Wilms H. Sulzer D. (2003). Neuromelanin of the substantia nigra: a neuronal black hole with protective and toxic characteristics. *Trends Neurosci.* 26 578–580. 10.1016/j.tins.2003.08.009 14585596

